# Consensuses and controversies on pseudomyxoma peritonei: a review of the published consensus statements and guidelines

**DOI:** 10.1186/s13023-021-01723-6

**Published:** 2021-02-13

**Authors:** Yu-Lin Lin, Da-Zhao Xu, Xin-Bao Li, Feng-Cai Yan, Hong-Bin Xu, Zheng Peng, Yan Li

**Affiliations:** 1 Department of Peritoneal Cancer Surgery, Beijing Shijitan Hospital, Capital Medical University, No. 10 Tieyi Road, Yangfangdian Street, Haidian District, Beijing, 100038 China; 2 Department of Pathology, Beijing Shijitan Hospital, Capital Medical University, Beijing, 100038 China; 3 Department of Myxoma, Aero Space Central Hospital, Peking University, Beijing, 100049 China; 4 Department of General Surgery, Chinese PLA General Hospital, Beijing, 100853 China

**Keywords:** Pseudomyxoma peritonei, Clinical management, Peritoneal Surface Oncology Group International, Consensus, Controversy, Peritoneal carcinomatosis, Traditional narrative review

## Abstract

**Background:**

Pseudomyxoma peritonei (PMP) is a clinical malignant syndrome mainly originating from the appendix, with an incidence of 2–4 per million people. As a rare disease, an early and accurate diagnosis of PMP is difficult. It was not until the 1980s that the systematic study of this disease was started.

**Main body:**

As a result of clinical and basic research progress over the last 4 decades, a comprehensive strategy based on cytoreductive surgery (CRS) + hyperthermic intraperitoneal chemotherapy (HIPEC) has been established and proved to be an effective treatment for PMP. Currently, CRS + HIPEC was recommended as the standard treatment for PMP worldwide. There are several consensuses on PMP management, playing an important role in the standardization of CRS + HIPEC. However, controversies exist among consensuses published worldwide. A systematic evaluation of PMP consensuses helps not only to standardize PMP treatment but also to identify existing controversies and point to possible solutions in the future. The controversy underlying the consensus and vice versa promotes the continuous refinement and updating of consensuses and continue to improve PMP management through a gradual and continuous process. In this traditional narrative review, we systemically evaluated the consensuses published by major national and international academic organizations, aiming to get a timely update on the treatment strategies of CRS + HIPEC on PMP.

**Conclusion:**

Currently, consensuses have been reached on the following aspects: pathological classification, terminology, preoperative evaluation, eligibility for surgical treatment, maximal tumor debulking, CRS technical details, and severe adverse event classification system. However, controversies still exist regarding the HIPEC regimen, systemic chemotherapy, and early postoperative intraperitoneal chemotherapy.

## Background

Pseudomyxoma peritonei (PMP) is a rare malignant clinical syndrome with an incidence of 2–4 per million people [1, 2]. The main feature of PMP is the extensive dissemination of copious mucus-containing tumor cells in the abdominal cavity. Mucus accumulation causes progressive abdominal distention, intestinal obstruction, malnutrition, cachexia, and ultimately death. As a rare disease, an early and accurate diagnosis of PMP is difficult, which often leads to the clinically advanced stage at the time of optimal clinical treatment.

**Fig. 1:**
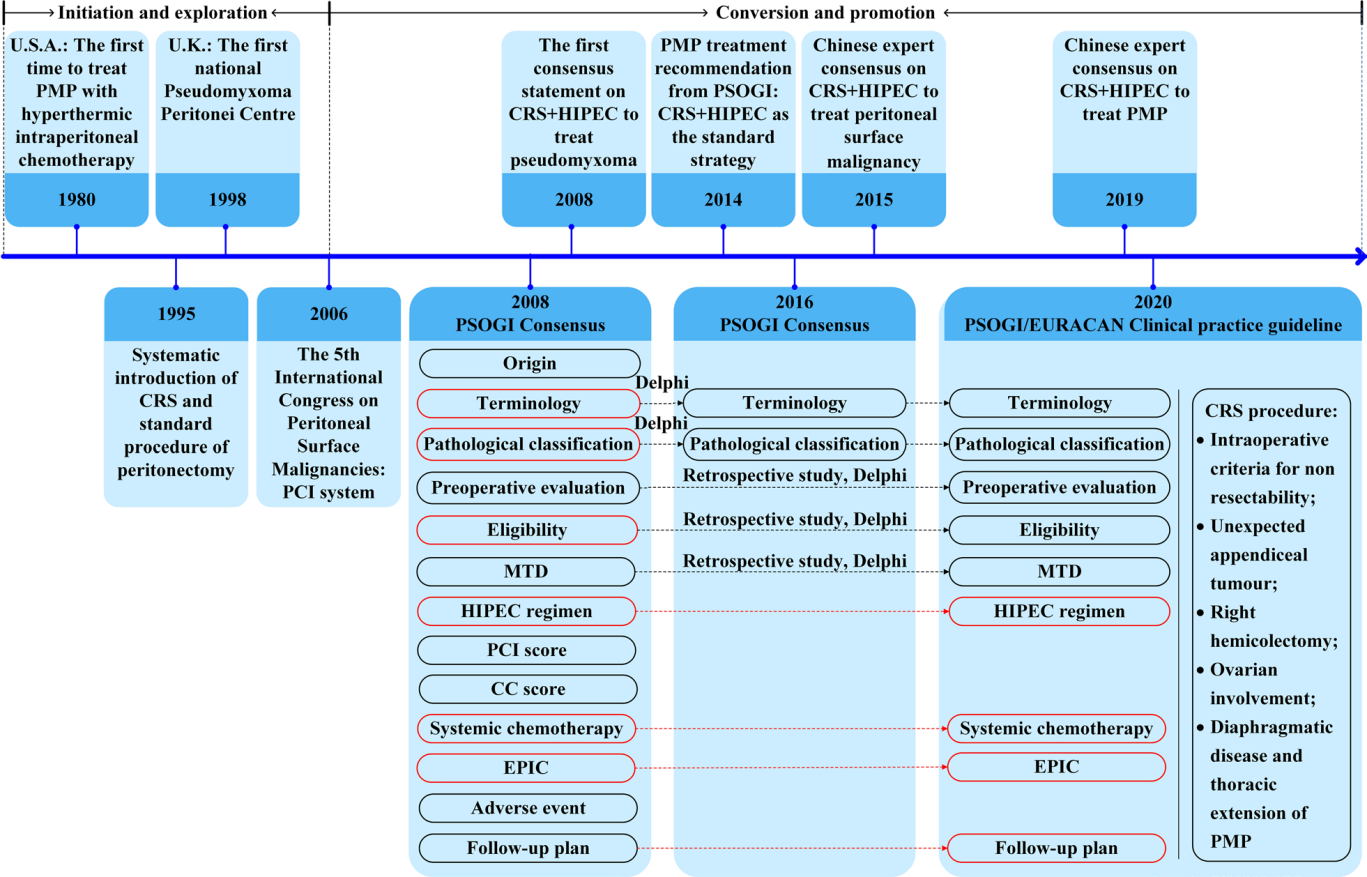
The historical development of pseudomyxoma peritonei (PMP) management and consensuses and controversies among different clinical recommendations worldwide. Consensus, black frame; Controversy, red frame. PSOGI, Peritoneal Surface Oncology Group International; EURACAN, European Rare Cancer; PMP, pseudomyxoma peritonei; MTD, maximal tumor debulking; HIPEC, hyperthermic intraperitoneal chemotherapy; CC, completeness of cytoreduction; EPIC, early postoperative intraperitoneal chemotherapy; CRS, cytoreductive surgery

However, the dilemma has been gradually solved since 1980s, when cytoreductive surgery (CRS) + hyperthermic intraperitoneal chemotherapy (HIPEC) strategy was developed [3]. In the following 4 decades, CRS + HIPEC related studies were discussed at the International Congress on Peritoneal Surface Malignancies since 1998, producing several consensus statements and guidelines (Fig. 1). Currently, CRS + HIPEC is the recommended treatment for PMP, vastly enhancing the prognosis of patients.

Although several guidelines have been published, most of the data come from retrospective studies, producing only type 3 clinical evidence. As a result, there are still many controversies regarding the treatment of PMP, such as the HIPEC regimen, efficacy of systemic chemotherapy, and early postoperative intraperitoneal chemotherapy (EPIC).

In this review, we collected literature and consensus statements or guidelines on PMP published worldwide over the past 4 decades, aiming to summarize the consensuses and controversies in PMP clinical management and to better understand the difficulties in the clinical management of PMP.

## Main text

### Literature search method

**Fig. 2:**
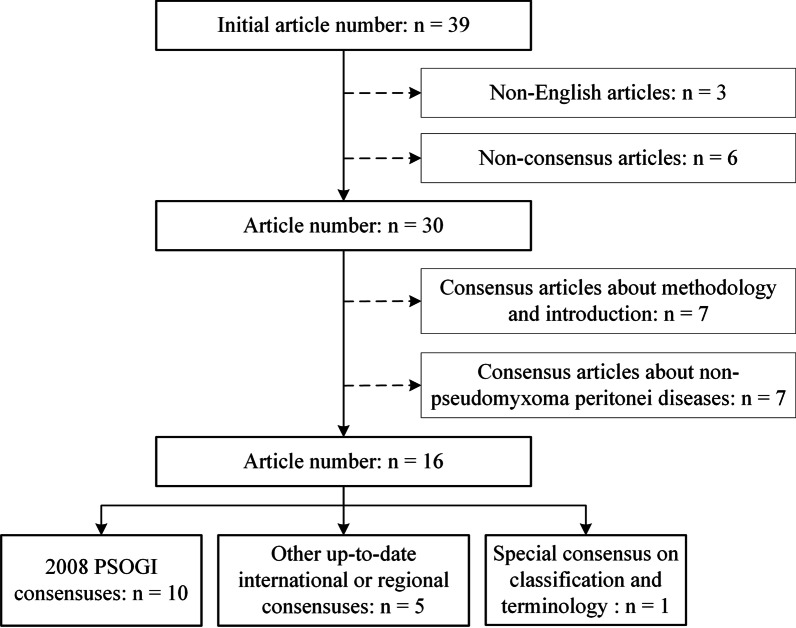
Flowchart of literature search process

We conducted traditional narrative review on the published PMP guidelines and consensuses, according to the methodology of a traditional narrative review [4, 5]. Figure 2 shows the selection process of this study. Literature search was performed using PubMed and Web of Science for published English literature. The search terms included “pseudomyxoma peritonei + guideline”, “pseudomyxoma peritonei + consensus”, “pseudomyxoma peritonei + PSOGI”, “pseudomyxoma peritonei + protocol”, and “pseudomyxoma peritonei + proposal”. To ensure the study quality, only consensus articles published by international or regional authoritative organizations were selected for analyses, including Peritoneal Surface Oncology Group International (PSOGI)/European Rare Cancer (EURACAN), Chicago Consensus Working Group (CCWG) from U.S.A., Chinese Anti-Cancer Association (CACA) from China; Latin American Registry of Peritoneal Diseases (LARPD) from Latin America; and Brazilian Society of Surgical Oncology (BSSO) from Brazil. Eventually, 39 articles were found and 16 were included in this study, including 10 articles from 2008 PSOGI consensuses, 1 special consensus on classification and terminology, and 5 international/regional consensuses. All these articles were analyzed and outlined according to the clinical diagnosis and treatment process of PMP, mainly following the logic of CRS + HIPEC. After synthesizing the studies, we provided a take-home-message paragraph to summarize the key points of this narrative review.

### Development of consensus statements worldwide

Since the first consensus statement by the PSOGI was published in 2008, sequential HIPEC treatment after CRS has gradually become the standard treatment of PMP in many countries or regions. There have been several different versions of consensuses on PMP customized according to actual situations in different regions. Currently, there are 5 up-to-date consensuses in different regions, coming from PSOGI/EURACAN, CCWG (U.S.A.) [6], CACA (China) [7], LARPD (Latin America) [8], and BSSO (Brazil) [9].

As listed in Table 1, the greatest differences among the 5 consensuses are the exclusion criteria, HIPEC regimen, and systemic chemotherapy, which still exist in the PSOGI/EURACAN 2020 Guideline. Consensuses on the terminology, pathological classification, and preoperative evaluation are largely identical with only minor differences, similar to the PSOGI/EURACAN 2020 Guideline. However, only 1 consensus at most provided recommendations regarding maximal tumor debulking, EPIC, and adverse events. The follow-up plan also needs further exploration. It is obvious that the 2008 and 2016 PSOGI Consensuses had a significant impact on consensus making around the world, which implies the important leading role of the PSOGI in PMP management. Due to the promotion and influence of the PSOGI, more countries have begun to summarize their experiences with PMP treatment [10].

**Table 1:** Consensus statements or guidelines worldwide

Items	Consensus statements or guidelines				
	PSOGI/EURACAN 2020 Guideline	2020 CCWG, U.S.A	2019 CACA, China	2018 LARPD, Latin America	2017 BSSO, Brazil
Terminology	The same as the 2016 PSOGI Consensus	NA	2016 PSOGI Consensus	(1) DPAM and PMCA-I/D: could be used; (2) LMCP and HMCP: better classification	2016 PSOGI Consensus or 7th AJCC staging system
Pathological classification	The same as the 2016 PSOGI Consensus	WHO Classification of Tumors, 5th edition	2016 PSOGI Consensus or 7th AJCC staging system	NA	2016 PSOGI Consensus or 7th AJCC staging system
*Patient selection*					
Preoperative evaluation	(1) Serum tumor markers: CEA and CA 199: should be always performed; CA 125: could be considered; (2) Cross sectional imaging: CT: should be always performed; MRI: could be considered; (3) Colonoscopy: should be always performed; (4) Laparoscopic evaluation: could be considered (5) Histological diagnostic confirmation: could be considered; (6) Core needle biopsy or explorative laparoscopy: could be considered (7) Histological review: should be always performed	(1) Preferred imaging modality: CT and MRI; (2) Staging of advanced and/or recurrent low-grade mucinous tumors and high-grade tumors: additional chest CT;	(1) Serological examination: CEA, CA125 and CA199; (2) CT examination is the optimal choice for routine recommendation; (3) gastrointestinal iodine water radiography; (4) Laparoscopic exploration and exfoliative cytology as optional examinations	(1) CT scan: fundamental examination; (2) MRI: useful tool; (3) Laparoscopy when imaging exams are not able to define the extent of disease; (4) Evaluation of extraperitoneal metastases: CT scan and PET-CT being fundamental exams. MRI is a useful tool	(1) Physical examination; (2) Cardiopulmonary investigation; (3) Renal function investigation; (4) Biological evaluation of hepatic function; (5) Nutritional state by BMI; (6) Extent of disease and staging by contrast-enhanced multi-sliced CT; (7) Additional FDG-PET, MRI or laparoscopy exploration if necessary
Exclusion criteria	Contra-indications: (1) Age > 75 years: relative; (2) Aggressive histologies (e.g. high-grade PMP with SRC, mucinous adenocarcinoma with SRC, GCC) and PCI > 20: relative; (3) Extensive small bowel serosal involvement: absolute; (4) Mesenteric involvement causing retraction: absolute; (5) Involvement of liver hilum: relative; (6) Infiltration of the lesser sac: relative; (7) Ureteric obstruction: relative; (8) Need for complete gastric resection: relative;	NA	(1) Distant organ metastasis during the preoperative assessment; (2) Serum bilirubin, aspartic aminotransferase and alanine aminotransferase > 2 ULN; (3) Serum creatinine > 1.2 ULN; (4) Moderate-severe contraction of the mesentery by an imaging examination; (5) Absolute contraindications to a routine operation	(1) ECOG Performance Status—PS 0 or 1; (2) Ages up to 75 years; (3) BMI should not be a contraindication; (4) Hemodynamic instability; (5) Coagulation disorder; (6) Surgical complications	(1) Extra-abdominal metastasis; (2) Massive involvement of the small bowel and its mesentery; (3) Hepatic pedicle and gastro-hepatic ligament, gross retroperitoneal lymph node involvement; (4) Ureteral or biliary obstruction; (5) PCI cut-off value (i.e.: PCI > 20) should not be applied as an absolute exclusion criterion
Maximal tumor debulking	Could be considered	Discouraged except for selected patients with low-grade tumors	NA	NA	NA
HIPEC regimen	Consensus not reached	(1) Mitomycin, 30 mg at time 0 min and 10 mg at time 60 min, 90 min (2) Mitomycin at 30 mg/m^2^ for 90 to 120 min (3) Mitomycin 15 mg/m^2^ + doxorubicin 15 mg/m^2^, 90 min (4) Oxaliplatin 300 mg/m^2^, 30 min	Docetaxel 120 mg or mitomycin C 30 mg + cisplatin 120 mg, 60 min	(1) Mitomycin C 35 mg/m^2^, 60-90 min, total intraperitoneal dose ≤ 70 mg; (2) Oxaliplatin 360–460 mg/m^2^, 30 min, combined with intravenous chemotherapy (5-FU 400 mg/m^2^ + LV 20 mg/m^2^)	(1) Oxaliplatin 360 mg/m^2^, 30 min at 4 L of perfusate; (2) CDDP 100 mg/m^2^ plus doxorubicin 15 mg/m^2^, 60 min at 4 L of perfusate
Intraoperative evaluation	PCI score	PCI score	PCI score	PCI score	PCI score
Residue evaluation	CC score	CC score	CC score	CC score	CC score
*Systemic chemotherapy*					
Adjuvant chemotherapy	Could be considered; The chemotherapy regimen should ideally consist of a combination of a fluoropyrimidine and an alkylating agent	A part of multimodality therapy for HMCP	(1) Acellular mucin and LMCP, surveillance; (2) HMCP and HMCP-S: 5-FU based regimen (e.g.: FOLFOX or FOLFIRI); (3) 6 cycles recommended	Suitable for high-grade tumor after optimal CRS + HIPEC	Guided by stands for other advanced colorectal cancers
Neoadjuvant chemotherapy	Could be considered; The chemotherapy regimen should ideally consist of a combination of a fluoropyrimidine and an alkylating agent	Considerable (a total of 6 months)	NA	No role even in high-grade tumor	Option for high-grade peritoneal metastasis from appendiceal adenocarcinoma with signet ring cells and moderate to high PCI scores
Palliative chemotherapy	Could be considered; The chemotherapy regimen should ideally consist of a combination chemotherapy together with a neo-angiogenesis inhibitor (e.g. bevacizumab)	Unresectable disease and/or recurrent disease not amenable to resection: according to treatment algorithms in mucinous colorectal cancer	NA	NA	NA
EPIC	Could be considered	NA	NA	NA	Optional when HIPEC is not available
Adverse event	NA	NA	NA	NA	(1) 2008 PSOGI Consensus; (2) Reported in Clavien-Dindo classification as well
*Follow-up plan*					
Frequency	(1) During the first 2 years Physical examination and thoracic, abdominal, and pelvic CT scan: every 6 months; (2) From 2 years onward: Physical examination: consensus not reached; Thoracic, abdominal, and pelvic CT scan: yearly; (3) Tumor markers: every 6 months	NA	(1) First 2 years: every 3 months; (2) Third year: every 6 months; (3) Fourth years and onward: annually;	NA	NA
Main contents	(1) Physical examination; (2) Thoracic, abdominal, and pelvic CT scan; (3) Tumor markers	NA	(1) Physical examination; (2) Serum tumor markers, including CEA, CA199, and CA125; (3) Contrast-enhanced CT + 3D reconstruction of the chest, abdomen, and pelvis	CEA used in 65.3% of the centers surveyed	NA

PSOGI, Peritoneal Surface Oncology Group International; EURACAN, European Rare Cancer; CCWG, Chicago Consensus Working Group; CACA, Chinese Anti-Cancer Association; LARPD, Latin American Registry of Peritoneal Diseases; BSSO, Brazilian Society of Surgical Oncology; NA, not applicable; GCC, goblet cell carcinoid; DPAM, disseminated peritoneal adenomucinosis; PMCA-I/D, peritoneal mucinous carcinomatosis with intermediate or discordant feature; LMCP, low-grade mucinous carcinoma peritonei; HMCP, high-grade mucinous carcinoma peritonei; AJCC, American Joint Committee on Cancer; WHO, World Health Organization; CT, computed tomography; MRI, magnet resonance imaging; PET, positive emission tomography; BMI, body mass index; FDG, β-2-[18 F]-Fluoro-2-deoxy-D-glucose; ULN, upper limits of normal; CDDP, cisplatin; ECOG, Eastern Cooperative Oncology Group; LV, leucovorin; PCI, peritoneal cancer index; CC, completeness of cytoreduction; PMCA-S, peritoneal mucinous carcinomatosis with signet ring cells; CRS, cytoreductive surgery; HIPEC, hyperthermic intraperitoneal chemotherapy; EPIC, early postoperative intraperitoneal chemotherapy; CEA, carcinoembryonic antigen; CA 199, carbohydrate antigen 199; CA 125, carbohydrate antigen 125

### Consensus on diagnosis and treatment

#### Consensus on the pathological diagnosis and classification

PMP pathological classification criteria and diagnostic terminology are confusing because there are multiple classification systems in the world. This confusion is indicative of the diverse clinical manifestations, variable pathological characteristics, and elusive features of PMP. Only through long-term and painstaking research can we properly understand its key pathogenesis and mechanism of progression before formulating appropriate clinical prevention and treatment strategies. The commonly used pathological classification methods in the literature include the Ronnett three-tier system [11], the Bradley two-tier system [12], and the WHO two-tier system [13]. The simultaneous use of different classification systems may have the following disadvantages: (1) the research results of different centers are heterogeneous and are thus not conducive to the comparison of identical or similar studies; (2) as a rare disease, it is not conducive to the organization of relatively scarce research resources for collaborative studies; (3) Ronnett's three-tier system includes non-appendiceal PMP; and (4) both the Bradley and WHO systems leave out the classification of signet ring cells. Because of these shortcomings, the PSOGI failed to reach a consensus on the PMP pathology classification in 2008 [14].

**Fig. 3:**
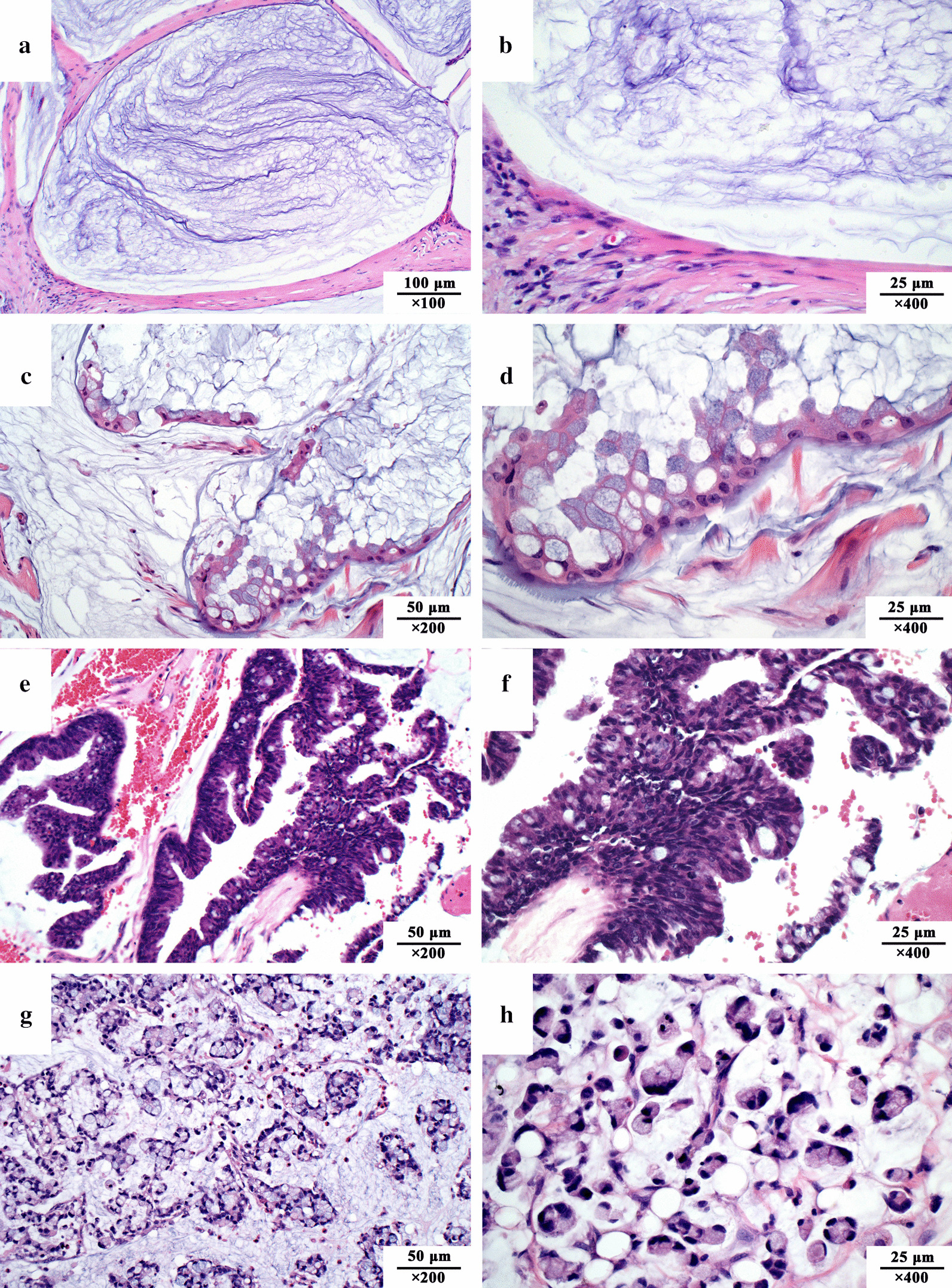
Pathological classification of pseudomyxoma peritonei in the 2016 consensus of the Peritoneal Surface Oncology Group International. **a**, **b**: Acellular mucin, without identifiable tumor cells in the disseminated peritoneal mucinous deposits; **c**, **d** Low-grade mucinous carcinoma peritonei, with tumor cells forming band-, island-, wave- or cluster-shaped tissue. Cancer cells present with a monolayer or pseudostratified arrangement, with slight nucleus atypia and rare mitotic figures; **e**, **f** High-grade mucinous carcinoma peritonei, with a complex structure presenting band-, island-, gland-, cribriform-shaped tissue, abundant cellularity, and at least local regional severe atypia; **g**, **h** High-grade mucinous carcinoma peritonei with signet ring cells, with abundant signet ring cells floating in the mucous pools. All sections were stained with H&E

An expert consensus on the pathological classification and diagnostic terms of PMP is particularly important (Table 2, Fig. 3), as it not only relates to the diagnosis and prognosis of PMP but also determines the treatment strategy. The fundamental treatment principle of PMP is to adopt different treatment strategies for PMP of different pathological grades. At the 12th International Conference on Peritoneal Carcinoma in Berlin in 2012, experts had heated discussions on PMP pathological classification and diagnostic terminology. It was not until 2016 that a written consensus on PMP pathology classification and diagnostic terminology was published [15]. According to this consensus, PMP is divided into 4 categories: (1) acellular mucin; (2) low-grade mucinous carcinoma peritonei (LMCP) or disseminated peritoneal adenomucinosis (DPAM); (3) high-grade mucinous carcinoma peritonei (HMCP) or peritoneal mucinous carcinomatosis (PMCA); and (4) high-grade mucinous carcinoma peritonei with signet ring cells (HMCP-S) or peritoneal mucinous carcinomatosis with signet ring cells (PMCA⁃S). It should be noted that DPAM and PMCA are synonyms for LMCP and HMCP, respectively, which are no longer recommended as standard pathological terminology [16].

**Table 2:** The 2016 PSOGI, 2017 AJCC, and 2019 WHO pathological classification and terminology of the PMP

2016 PSOGI classification	Counterparts	
	2017 AJCC staging system, 8th edition (TNM)	2019 WHO classification of tumors, 5th edition
**AM:** (1) Mucin without neoplastic epithelium; (2) Confined to or distant from organ surface	**M1a**	**pM1a**
**LMCP:** (1) Low-grade cytology; (2) Rare mitosis; (3) Few tumoral mucinous epithelium (< 20% of tumor volume)	**M1b. G1,** well-differentiated	**pM1b, Grade 1:** (1) Hypocellular mucinous deposits; (2) Neoplastic epithelial elements have low-grade cytology; (3) No infiltrative-type invasion
**HMCP:** *Features of one or more of the following (At least focally):* (1) high-grade cytology; (2) Infiltration of adjacent tissues; (3) Invasion of vascular lymphatic vessels or surrounding nerves; (4) Cribriform growth; (5) Neoplastic mucinous epithelium (> 20% of tumor volume); *Sub-classification based on differentiation* (1) well-differentiated: Mainly composed of single- tubular glands; Tumor cell polarity exists; Obvious tumor cell atypia; Infiltrative components; (2) Moderately-differentiated: Solid sheet tumor cells mixed with adenoid structures; Minimal or no polarity; (3) Poorly-differentiated: Highly irregular to no adenoid differentiation Cell polarity disappears	**M1b. G2 or G3,** moderately- or poorly-differentiated	**pM1b, Grade 2:** (1) Hypercellular mucinous deposits as judged at 20 × magnification; (2) High-grade cytological features; (3) Infiltrative-type invasion characterized by jagged or angulated glands in a desmoplastic stroma, or a small mucin pool pattern with numerous mucin pools containing clusters of tumor cells
**HMCP-S:** Tumor with signet ring cell component (signet ring cells ≥ 10%)	**M1b. G3,** poorly- differentiated; PMCA-S	**pM1b,** Mucinous tumor deposits with signet-ring cells

PMP, pseudomyxoma peritonei; PSOGI, Peritoneal Surface Oncology Group International; AJCC, American Joint Committee on Cancer. AM, acellular mucin; LMCP, low-grade mucinous carcinoma peritonei; HMCP, high-grade mucinous carcinoma peritonei; HMCP-S, high-grade mucinous carcinoma peritonei with signet ring cells; DPAM, disseminated peritoneal adenomucinosis; PMCA-I, peritoneal mucinous carcinomatosis with intermediate feature; PMCA, peritoneal mucinous carcinomatosis; NA, not applicable

In 2017, the 8th Edition of the American Joint Committee on Cancer (AJCC) Cancer Staging Manual included the intraperitoneal dissemination of acellular mucin in appendix mucinous tumors in M1a, while the intraperitoneal dissemination containing cellular mucin was divided into M1b. The AJCC further divided M1b into 3 grades: (1) G1, well-differentiated mucinous tumors; (2) G2, moderately differentiated mucinous tumors; and (3) G3, poorly differentiated mucinous tumors. In 2019, WHO published another taxonomy similar to 2017 AJCC staging system, and 2016 PSOGI classification (Table 2).

Currently, the 2016 PSOGI classification system is widely recognized by peritoneal carcinomatosis (PC) experts around the world. The latter-developed two taxonomies, the 2017 AJCC staging system and 2019 WHO classification of tumor, are similar to the 2016 PSOGI taxonomy in the classification criteria. However, it must be realized that the significance of the 2016 PSOGI Consensus is to end controversies regarding PMP pathology classification and diagnostic terminology. The relationship between the PSOGI pathological grading and outcome stratification still requires further study [17].

#### Consensus on the preoperative evaluation

**Fig. 4:**
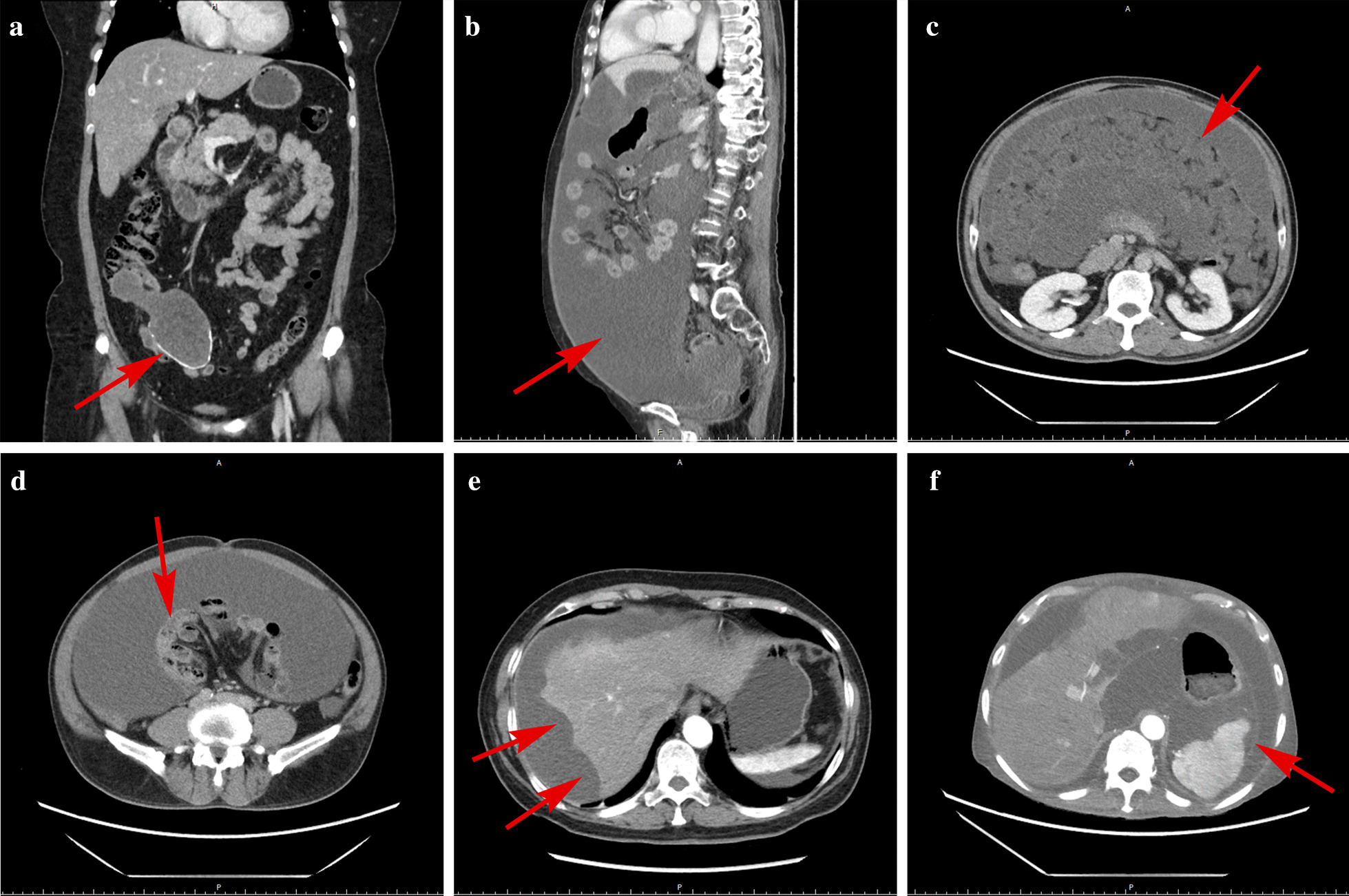
Typical computed tomography characteristics of pseudomyxoma peritonei. Computed tomography shows the following: **a** Enlargement of the appendiceal cavity and calcification of the appendiceal wall; **b** Abdominal girth enlargement caused by a large volume of intraperitoneal mucus deposits presenting as a “jelly belly”; **c** Thickened greater omentum presenting as an “omental cake”; **d** Small intestines compressed by mucus causing “central displacement”; **e** Scallop impression on the surface of the liver; **f** Contour deformation of the spleen

A consensus on the preoperative evaluation for PMP was reached in the 2008 PSOGI Consensus, which greatly facilitated patient diagnosis and selection, mainly including 4 aspects [7]. (1) Serum tumor markers, which mainly combined testing of carcinoembryonic antigen (CEA), carbohydrate antigen 125 (CA125), and carbohydrate antigen 199 (CA199). CEA, CA125, and CA199 are helpful indicators for evaluating the degree of tumor invasion, ascites production and tumor burden, and the proliferation of cancer cells, respectively. (2) A computed tomography (CT) examination + 3D reconstruction is the optimal choice for routine preoperative examination. Typically, CT scan of PMP revealed a right lower abdominal cystic or cystic-solid mass frequently with calcification (Fig. 4a); copious mucinous ascites in the abdominal cavity (Fig. 4b); extensive organ invasion or compression (Fig. 4c–f); (3) Laparoscopic exploration and exfoliative cytology are both optional.

#### Consensus on the intraoperative evaluation

**Fig. 5:**
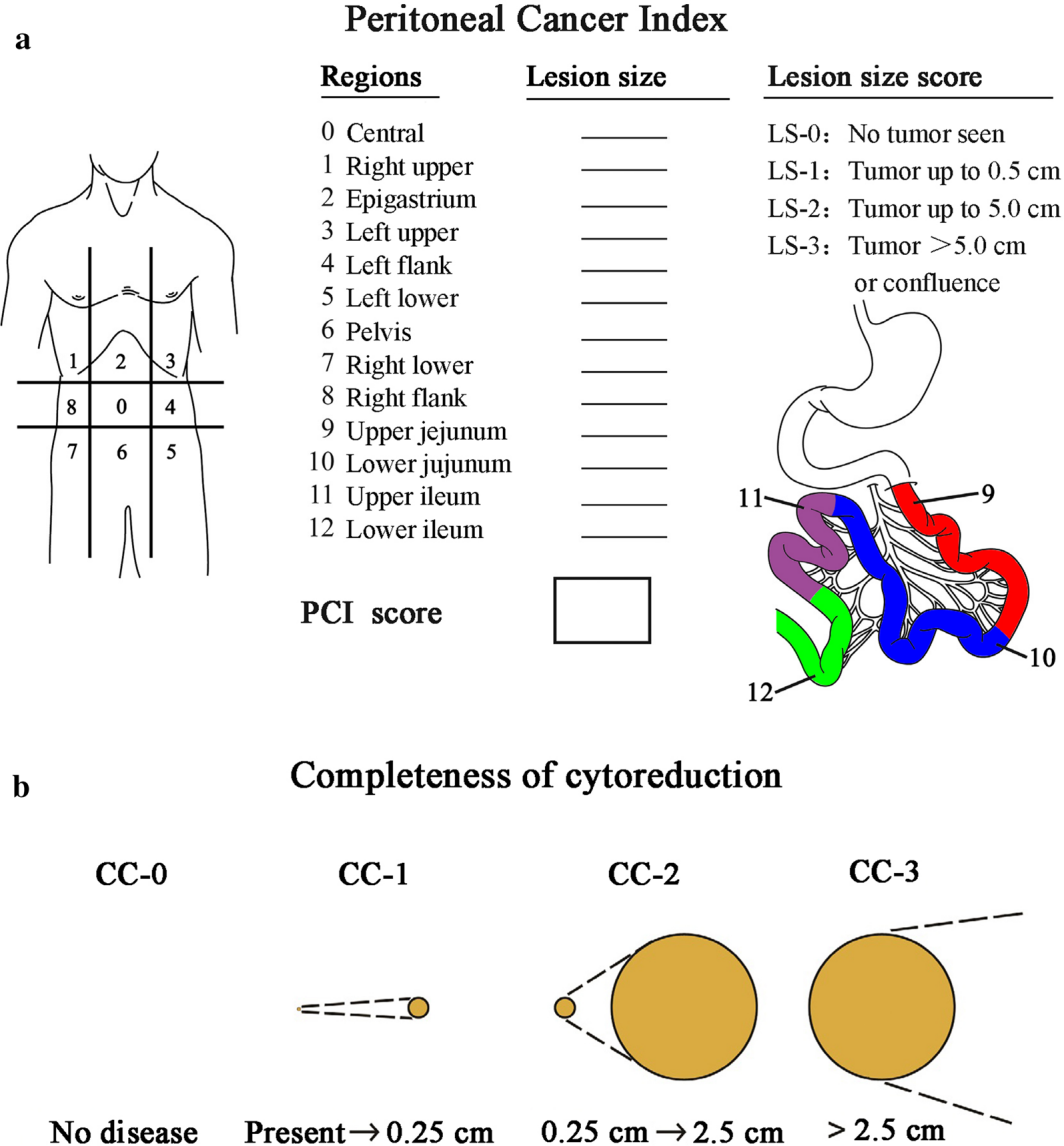
Scoring criteria of the intraoperative peritoneal cancer index and postoperative completeness of cytoreduction. **a** peritoneal cancer index score; **b** completeness of cytoreduction score

The peritoneal cancer index (PCI) score is a standard parameter used to evaluate tumor burden during comprehensive abdominal exploration. According to the Sugarbaker standard PCI score [18], the abdomen is divided into 13 areas (Fig. 5a), including 9 abdominopelvic regions and 4 additional regions in the small intestine. Lesion size (LS) is scored according to the following rules: LS-0, no visible tumor; LS-1, tumor diameter ≤ 0.5 cm; LS-2, tumor diameter 0.5–5.0 cm; and LS-3, tumor diameter > 5.0 cm or confluence. The total score of the 13 regions ranges from 0 to 39 points. The PCI scoring system helps to evaluate tumor load in the abdominal cavity and has important significance for confirming regions in the peritoneum that need to be removed or stripped or whether an optimal CRS can be performed. A high PCI score is an independent factor for poor PFS [19].

The completeness of cytoreduction (CC) score (Fig. 5b) is the main prognostic factor for PC patients. It is suitable for PMP, colon cancer peritoneal metastasis, peritoneal sarcomatosis, peritoneal malignant mesothelioma, and ovarian cancer peritoneal metastasis [20]. The CC scoring standard has become not only an objective quantitative index and independent prognostic factor for evaluating the effect of tumor resection but also an important part of the standardized CRS. The specific evaluation is as follows: CC-0, no residual tumor nodule after cytoreduction; CC-1: residual tumor diameter < 2.5 mm; CC-2: residual tumor diameter 2.5 mm-2.5 cm; and CC-3: residual tumor diameter > 2.5 cm or the residual tumor cannot be removed or palliatively removed.

#### Consensus on the standard operating procedures of CRS + HIPEC

**Fig. 6:**
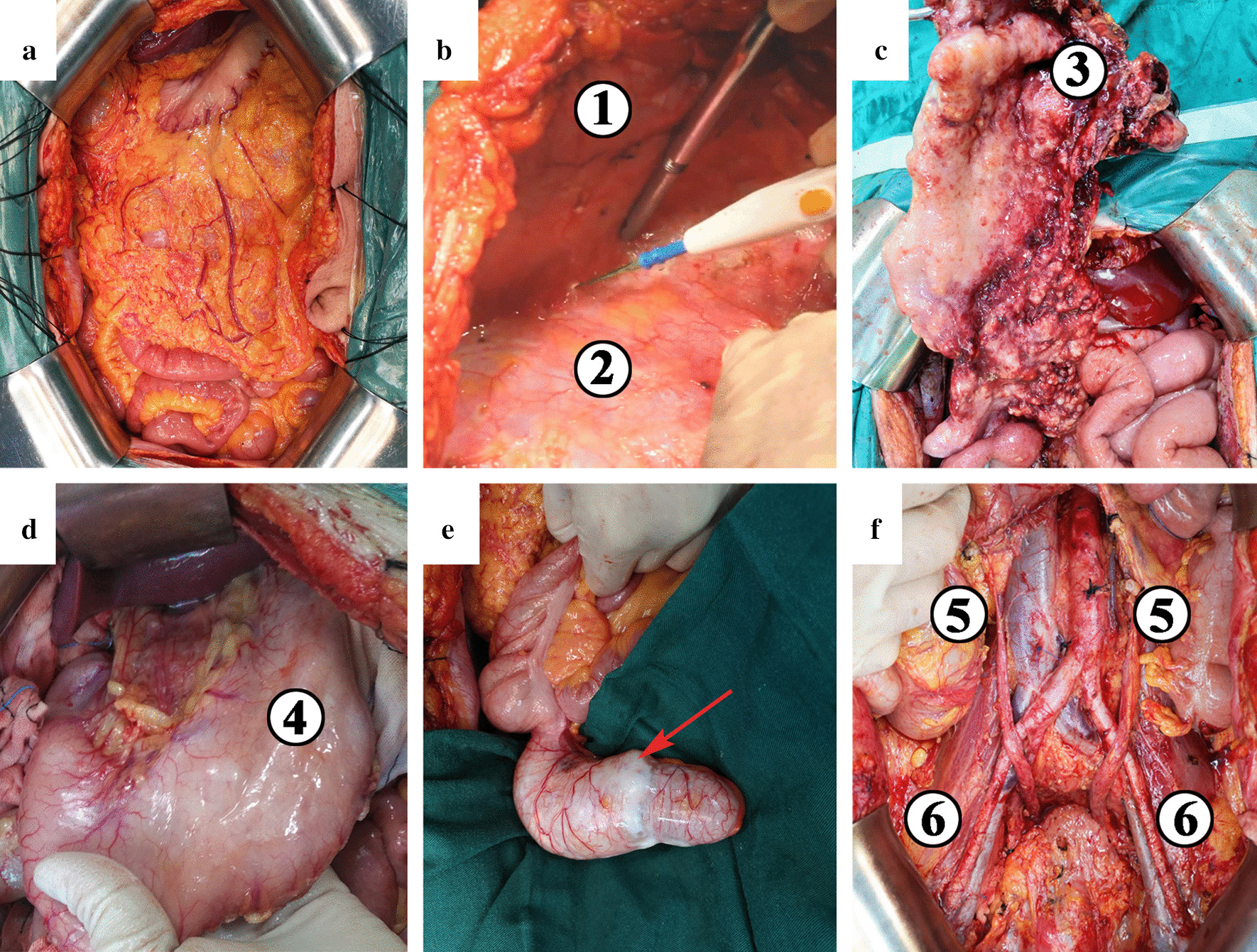
Main procedures of cytoreductive surgery. **a** A midline incision from xiphoid to pubic symphysis; **b** Total anterior parietal peritonectomy. ① Right abdominal wall. ② Right parietal peritoneum. **c** Greater omentectomy. ③ Greater omentum; **d** Exploration of the lesser omentum and stomach. ④ Stomach; **e** Appendectomy. The appendix significantly distended with serosa infiltrated; **f** Complete pelvic peritonectomy. ⑤ Ureter. ⑥ External iliac artery

The implementation of standardized CRS and complete resection of all visible malignant tumors is the basis for long-term survival. Sugarbaker elaborated on the PMP peritonectomy procedure as early as 1995 [21]. Complete CRS may require a 6-step peritonectomy to completely remove all tumors implanted on the peritoneum. These 6 procedures include greater omental excision + splenectomy; left upper peritoneal resection; right upper peritoneal resection; lesser omental excision + cholecystectomy + omental bursa peritonectomy; pelvic peritonectomy + sleeve resection of the sigmoid colon; and antrectomy. In 2003, Sugarbaker further improved the surgical principles and technical specifications for peritoneal resection in the pelvic peritoneum, left upper peritoneum, right upper peritoneum, greater omentum + spleen, and lesser omentum + gallbladder [22]. After 4 decades’ clinical practice, the surgical procedures and details have been refined and standardized, which are accepted by PC centers all over the world (Fig. 6).

#### Consensus on the perioperative management

When PMP patients receive CRS + HIPEC, perioperative safety management is of paramount importance. The main high-risk factors for PMP patients include heavy tumor burden, multiple previous operations and chemotherapies, long anesthesia and operation time, extensive resections, high-volume bleeding and blood infusion, and central venous catheter. The main adverse events include perioperative venous thrombosis, anastomotic leakage, bleeding, infection, and postoperative hypermyoglobinemia. Therefore, the clinical consensus on the perioperative management of CRS + HIPEC has also become important for preventing complications. The most important aspect of perioperative management is the graded evaluation system of adverse events. The current consensus is that the perioperative period of peritoneal surface tumors includes 9 categories, 48 adverse events [23, 24] (Additional file 1: Table S1) and the classification of grade I-IV adverse events as follows: Grade I, confirmed diagnosis but without intervention; Grade II, confirmed diagnosis requiring medical intervention; Grade III, confirmed diagnosis, conservative treatment, usually requires an imaging examination for disease evaluation; and Grade IV, definitive diagnosis, emergency intervention, reoperation or ICU treatment required.

### Controversies on the management of pseudomyxoma peritonei

Intraoperative procedures and technical details have been refined and standardized thanks to intensive collaborative efforts among PC centers around the world. The detailed and standardized procedures of peritonectomy and organ resection have been fully illustrated in *Cytoreductive Surgery & Perioperative Chemotherapy for Peritoneal Surface Malignancy: Textbook and Video Atlas* [25]. The published PC textbooks as well as the refinement and updating of expert consensuses have greatly contributed to the standardization of CRS. In contrast, several controversies remain to be solved regarding HIPEC regimens, such as drug choices, dosages, pharmacokinetics, and efficacies.

#### Controversies on HIPEC regimens

Controversies on HIPEC regimens have existed since their first application in 1980, and such controversies have inevitable negative impacts on the integration and comparison of clinical data and treatment efficacies among the PC centers. Among the frequently applied HIPEC regimens, most are based on oxaliplatin and mitomycin C. The representative oxaliplatin-based regimens are the “Elias high dose oxaliplatin regimen”, the “Glehen medium dose oxaliplatin regimen”, and the “Wake Forest University oxaliplatin regimen” (Table 3). However, considering the high rates of lethal hemorrhagic complications, lower-dosage oxaliplatin-based HIPEC regimens have been developed [26–29]. A consensus is almost impossible to reach due to the lack of high-level evidence from well-designed randomized controlled trials. Similarly, the dosage and intraperitoneal concentration of mitomycin C are also under heated debate [30, 31]. It is believed that the “Dutch High Dose Mitomycin C Regimen: ‘Triple Dosing Regimen’” is the preferred regimen for maintaining a stable intraperitoneal drug concentration. Other regimens, for example, the “Sugarbaker regimen” [32] and the “American Society of Peritoneal Surface Malignancy Low Dose Mitomycin C Regimen: ‘Concentration-Based Regimen’” [33], are also suggested by the PSOGI expert panel.

**Table 3:** Frequently used HIPEC regimens in the international peritoneal cancer centers

HIPEC regimens	Dosage (mg/m^2^)	Carrier solution	Duration (min)	HIPEC method	Remarks	Consensus for clinical trial (%)
*Oxaliplatin-based regimens*						
1. Elias high dose oxaliplatin regimen	460	2 L/m^2^, 5% dextrose solution	30	Open	Add 5-FU 400 mg/m^2^ and leucovorin 20 mg/m^2^ to separate bags of 250 mL normal saline. Begin rapid intravenous infusion of both drugs 1 h before intraperitoneal chemotherapy	8.9
2. Glehen medium dose oxaliplatin regimen	360	2 L/m^2^, 5% dextrose solution	30	Closed	Add 5-FU 400 mg/m^2^ and leucovorin 20 mg/m^2^ to separate bags of 250 mL normal saline. Begin rapid intravenous infusion of both drugs 1 h before intraperitoneal chemotherapy	28.6
3. Wake Forest University oxaliplatin regimen	200	3 L, 5% dextrose solution	120	Closed	NA	1.8
*Mitomycin C-based regimen*						
4. Sugarbaker regimen	Mitomycin C, doxorubicin: both 15 mg/m^2^	2 L, 1.5% dextrose peritoneal dialysis solution	90	Semi-open	Add 5-FU (400 mg/m^2^) and leucovorin (20 mg/m^2^) to separate bags of 250 mL normal saline. Begin rapid intravenous infusion of both drugs simultaneously with intraperitoneal chemotherapy	1.8
5. Dutch high dose mitomycin C regimen: “Triple Dosing Regimen”	35	3 L, 1.5% dextrose peritoneal dialysis solution	90	Semi-open	Add mitomycin C to the 1.5% peritoneal dialysis solution at a dose of 17.5 mg/m^2^ followed by 8.8 mg/m^2^ at 30 min and 8.8 mg/m^2^ at 60 min	42.9
6. American Society of Peritoneal Surface Malignancy low dose mitomycin C regimen: “Concentration-Based Regimen”	40 mg/3L	3 L, 1.5% dextrose peritoneal dialysis solution	90	Closed	Add mitomycin C to the 1.5% peritoneal dialysis solution at a dose of 30 mg/3 L followed by 10 mg at 60 min	14.3
7. PMI Basingstoke IP chemotherapy regimen: “Body Surface Area-based”	10	1 L sodium chloride 0.9%	60	Open	Consider dose reduction by 33% in case of following risk factors: a Obese (BMI > 40) b Severe abdominal distension c Prior heavy chemotherapy (last 3 months)	10.7
Others	NA	NA	NA	NA	NA	3.6

*HIPEC* hyperthermic intraperitoneal chemotherapy, *NA* not applicable

To date, HIPEC regimen-related studies have been mostly single- or multicenter large-sample analyses [17, 19, 34–48]. In 2018, Levine et al. published the first multicenter randomized controlled trial on appendix-derived PMP. The study compared the safety, quality of life (QOL), 3-year disease-free survival (DFS), and 3-year overall survival (OS) after HIPEC (oxaliplatin 200 mg/m^2^ *vs.* mitomycin C 40 mg) [49]. In terms of hematotoxicity, no significant differences in hemoglobin or platelet counts were found, while white blood cell counts were significantly lower in the mitomycin C group between postoperative days 5–10. Short-term QOL was similar, but the oxaliplatin group had higher scores regarding physical well-being and emotional well-being than the mitomycin group. In addition, no significant differences were found regarding the 3-year DFS and OS rates between the 2 groups.

#### Controversies on HIPEC methods

There is a consensus that the efficacy evaluation of different HIPEC regimens relies largely on randomized clinical trials. However, an unified HIPEC method, duration, and temperature are required to guarantee the accuracy and reliability of both multicenter clinical trials and retrospective analyses of large samples, which vary vastly among different centers (Table 3). The randomized trial conducted by Levine et al. provided valuable experience for HIPEC regimen-related clinical trials. With the strengthening cooperation among PC centers worldwide and the promotion of standardized CRS + HIPEC technology, a larger randomized clinical trial of higher quality might promote a HIPEC regimen with better efficacy and less toxicity. The PSOGI/EURACAN 2020 Guideline voted 2 favored regimens for clinical trials, i.e., the “Glehen Medium Dose Oxaliplatin Regimen” and the “Dutch High Dose Mitomycin C Regimen: ‘Triple Dosing Regimen’”. However, neither reached the consensus threshold (51.0%) (Table 3).

#### Controversies on EPIC

EPIC is usually performed from postoperative days 1 to 4/5 without heating and is easier to perform than HIPEC. Theoretically, EPIC has the advantage of reducing or even eliminating tumor cells trapped in fibrin deposition. Therefore, EPIC could be an adjuvant therapy to reduce postoperative recurrence when combined with HIPEC. Retrospective analyses have also supported that EPIC significantly prolongs the 5-year survival rate [50] and is an independent prognostic factor for prolonged DFS and OS [51, 52]. Despite the reported good efficacy of EPIC, its safety is unclear. Lam et al. [53] and Tan et al. [54] reported that CRS + HIPEC + EPIC increased postoperative adverse events. However, Huang and colleagues [51, 52] reported the opposite results. Considering safety issues and the lack of high-level clinical evidence, 37.5% of experts in the PSOGI/EURACAN 2020 Guideline did not recommend EPIC immediately after CRS + HIPEC, with 60.7% supporting EPIC. At present, one multicenter, prospective randomized clinical trial is being conducted at the Memorial Sloan Kettering Cancer Center to evaluate efficacy and toxicity between CRS + HIPEC and CRS + EPIC [55], the results of which might provide strong evidence for the efficacy and toxicity of EPIC.

#### Controversies on systemic chemotherapies

Systemic chemotherapy in PMP includes neoadjuvant systemic chemotherapy, adjuvant systemic chemotherapy, and palliative systemic chemotherapy. Although the level of evidence is low, the expert panel of the PSOGI/EURACAN 2020 Guideline reached a consensus on the application of palliative systemic chemotherapy in patients with unresectable tumors or who are not suitable for surgery. In addition, its combination with bevacizumab might contribute to prolonged PFS. With respect to neoadjuvant [19, 56–64] and adjuvant [57, 65–67] chemotherapy, no definite survival benefits were proven in low-grade PMP, high-grade PMP, or high-grade PMP with signet ring cells, which is quite controversial. According to the PSOGI expert panel, neoadjuvant or adjuvant chemotherapy should not be totally abandoned in low-grade PMP (92.7% and 74.5%, respectively) and can be considered in patients with high-grade PMP with signet ring cells (76.4% and 85.5%, respectively). If neoadjuvant or adjuvant systemic chemotherapy is needed, a combination of fluoropyrimidine and an alkylating agent (e.g., oxaliplatin) is recommended (87.3% for both).

## Conclusions

In conclusion, the up-to-date reached consensuses in PMP clinical management includes: (1) pathological classification; (2) terminology; (3) preoperative evaluation and eligibility for surgical treatment; (4) intraoperative evaluation; (5) standard CRS procedures and intraoperative criteria for non-resectability; and (6) SAE classification system. Gathering almost all PMP experts from all over the world, the PSOGI/EURACAN 2020 Guideline is the most authoritative clinical guideline for practice. At the same time, there are still several controversies existing: (1) HIPEC regimens; (2) systemic chemotherapy; and (3) EPIC.

PSOGI plays an important role in standardizing terminology and technical details, as well as in promoting exchanges and developing the PC discipline around the world. However, we should recognize that evidence of a consensus from the PSOGI is derived mainly from retrospective studies with low-level clinical evidence, and the Delphi methodology is not a substitution for randomized clinical trials. This is also the root cause for controversies regarding the HIPEC regimen, systemic chemotherapy, and EPIC. In the near future, consensuses or guidelines are still indispensable for the development in the management and study of PMP.

## Supplementary Information

**Additional file 1**. Classification of common adverse events during PMP perioperative period.

## Data Availability

Not applicable.

## Abbreviations

PMP: Pseudomyxoma peritonei
CRS: Cytoreductive surgery
HIPEC: Hyperthermic intraperitoneal chemotherapy
PSOGI: Peritoneal Surface Oncology Group International
EURACAN: European Rare Cancer
CCWG: Chicago Consensus Working Group
CACA: Chinese Anti-Cancer Association
LARPD: Latin American Registry of Peritoneal Diseases
BSSO: Brazilian Society of Surgical Oncology
LMCP: Low-grade mucinous carcinoma peritonei
DPAM: Disseminated peritoneal adenomucinosis
HMCP: High-grade mucinous carcinoma peritonei
PMCA: Peritoneal mucinous carcinomatosis
HMCP-S: High-grade mucinous carcinoma peritonei with signet ring cells
PMCA-S: Peritoneal mucinous carcinomatosis with signet ring cells
AJCC: American Joint Committee on Cancer
WHO: World Health Organization
CEA: Carcinoembryonic antigen
CA125: Carbohydrate antigen 125
CA199: Carbohydrate antigen 199
CT: Computed tomography
LS: Lesion size
CC: Completeness of cytoreduction
PC: Peritoneal carcinomatosis
QOL: Quality of life
DFS: Disease-free survival
OS: Overall survival

## References

[1] Smeenk RM, van Velthuysen ML, Verwaal VJ, Zoetmulder FA (2008). Appendiceal neoplasms and pseudomyxoma peritonei: a population based study. Eur J Surg Oncol.

[2] Mittal R, Chandramohan A, Moran B (2017). Pseudomyxoma peritonei: natural history and treatment. Int J Hyperthermia.

[3] Spratt JS, Adcock RA, Muskovin M, Sherrill W, McKeown J (1980). Clinical delivery system for intraperitoneal hyperthermic chemotherapy. Cancer Res.

[4] Reviews: from systematic to narrative: narrative review. https://guides.library.uab.edu/c.php?g=63689&p=409774.

[5] Narrative review. https://www.sciencedirect.com/topics/psychology/narrative-review.

[6] Chicago Consensus Working Group (2020). The Chicago consensus on peritoneal surface malignancies: management of appendiceal neoplasms. Cancer.

[7] Li Y, Xu HB, Peng Z, Cui SZ, Wu W (2019). Chinese expert consensus on cytoreductive surgery and hyperthermic intraperitoneal chemotherapy for pseudomyxoma peritonei. Natl Med J China.

[8] Latin American Registry of Peritoneal Diseases (2018). Current practice of Latin American centers in the treatment of peritoneal diseases with cytoreductive surgery with HIPEC. Eur J Surg Oncol.

[9] Batista TP, Sarmento BJQ, Loureiro JF, Petruzziello A, Lopes A, Santos CC (2017). A proposal of Brazilian Society of Surgical Oncology (BSSO/SBCO) for standardizing cytoreductive surgery (CRS) plus hyperthermic intraperitoneal chemotherapy (HIPEC) procedures in Brazil: pseudomixoma peritonei, appendiceal tumors and malignant peritoneal mesothelioma. Revista do Colegio Brasileiro de Cirurgioes.

[10] Bhatt A, Mehta S, Seshadri RA, Sethna K, Zaveri S, Rajan F (2016). The initial Indian experience with cytoreductive surgery and HIPEC in the treatment of peritoneal metastases. Indian J Surg Oncol.

[11] Ronnett BM, Zahn CM, Kurman RJ, Kass ME, Sugarbaker PH, Shmookler BM (1995). Disseminated peritoneal adenomucinosis and peritoneal mucinous carcinomatosis. A clinicopathologic analysis of 109 cases with emphasis on distinguishing pathologic features, site of origin, prognosis, and relationship to "pseudomyxoma peritonei". Am J Surg Pathol..

[12] Bradley RF, Stewart JH, Russell GB, Levine EA, Geisinger KR (2006). Pseudomyxoma peritonei of appendiceal origin: a clinicopathologic analysis of 101 patients uniformly treated at a single institution, with literature review. Am J Surg Pathol.

[13] Carr N, Sobin L, Bosman F, Carneiro C, Hruban R, Theise N (2010). Adenocarcinoma of the appendix. WHO classification of tumours of the digestive system.

[14] Moran B, Baratti D, Yan TD, Kusamura S, Deraco M (2008). Consensus statement on the loco-regional treatment of appendiceal mucinous neoplasms with peritoneal dissemination (pseudomyxoma peritonei). J Surg Oncol.

[15] Carr NJ, Cecil TD, Mohamed F, Sobin LH, Sugarbaker PH, Gonzalez-Moreno S (2016). A consensus for classification and pathologic reporting of pseudomyxoma peritonei and associated appendiceal neoplasia: the results of the Peritoneal Surface Oncology Group International (PSOGI) modified Delphi process. Am J Surg Pathol.

[16] Valasek MA, Pai RK (2018). An update on the diagnosis, grading, and staging of appendiceal mucinous neoplasms. Adv Anat Pathol.

[17] Baratti D, Kusamura S, Milione M, Bruno F, Guaglio M, Deraco M (2018). Validation of the recent PSOGI pathological classification of pseudomyxoma peritonei in a single-center series of 265 patients treated by cytoreductive surgery and hyperthermic intraperitoneal chemotherapy. Ann Surg Oncol.

[18] Portilla AG, Shigeki K, Dario B, Marcello D (2008). The intraoperative staging systems in the management of peritoneal surface malignancy. J Surg Oncol.

[19] Chua TC, Moran BJ, Sugarbaker PH, Levine EA, Glehen O, Gilly FN (2012). Early- and long-term outcome data of patients with pseudomyxoma peritonei from appendiceal origin treated by a strategy of cytoreductive surgery and hyperthermic intraperitoneal chemotherapy. J Clin Oncol.

[20] Fagotti A, Vizzielli G, Fanfani F, Costantini B, Ferrandina G, Gallotta V (2013). Introduction of staging laparoscopy in the management of advanced epithelial ovarian, tubal and peritoneal cancer: impact on prognosis in a single institution experience. Gynecol Oncol.

[21] Sugarbaker PH (1995). Peritonectomy procedures. Ann Surg.

[22] Sugarbaker PH (2003). Peritonectomy procedures. Surg Oncol Clin N Am..

[23] Sugarbaker PH, Alderman R, Edwards G, Marquardt CE, Gushchin V, Esquivel J (2006). Prospective morbidity and mortality assessment of cytoreductive surgery plus perioperative intraperitoneal chemotherapy to treat peritoneal dissemination of appendiceal mucinous malignancy. Ann Surg Oncol.

[24] Sugarbaker PH, Van der Speeten K, Stuart OA, Chang D, Mahteme H, Sugarbaker PH (2017). Patient- and treatment-related variables, adverse events and their statistical relationship for treatment of peritoneal metastases. Cytoreductive surgery & perioperative chemotherapy for peritoneal surface malignancy: textbook and video atlas.

[25] Bakrin N, Deraco M, Glehen O, Morris DL, Van der Speeten K (2017). Cytoreductive surgery & perioperative chemotherapy for peritoneal surface malignancy: textbook and video atlas, 2.

[26] Elias D, El Otmany A, Bonnay M, Paci A, Ducreux M, Antoun S (2002). Human pharmacokinetic study of heated intraperitoneal oxaliplatin in increasingly hypotonic solutions after complete resection of peritoneal carcinomatosis. Oncology.

[27] Pomel C, Ferron G, Lorimier G, Rey A, Lhomme C, Classe JM (2010). Hyperthermic intra-peritoneal chemotherapy using oxaliplatin as consolidation therapy for advanced epithelial ovarian carcinoma. Results of a phase II prospective multicentre trial. CHIPOVAC study. Eur J Surg Oncol..

[28] Chalret du Rieu Q, White-Koning M, Picaud L, Lochon I, Marsili S, Gladieff L (2014). Population pharmacokinetics of peritoneal, plasma ultrafiltrated and protein-bound oxaliplatin concentrations in patients with disseminated peritoneal cancer after intraperitoneal hyperthermic chemoperfusion of oxaliplatin following cytoreductive surgery: correlation between oxaliplatin exposure and thrombocytopenia. Cancer Chemother Pharmacol..

[29] Charrier T, Passot G, Peron J, Maurice C, Gocevska S, Quénet F (2016). Cytoreductive surgery combined with hyperthermic intraperitoneal chemotherapy with oxaliplatin increases the risk of postoperative hemorrhagic complications: analysis of predictive factors. Ann Surg Oncol.

[30] Mohamed F, Cecil T, Moran B, Sugarbaker P (2011). A new standard of care for the management of peritoneal surface malignancy. Curr Oncol.

[31] Levine EA, Stewart JH, Shen P, Russell GB, Loggie BL, Votanopoulos KI (2014). Intraperitoneal chemotherapy for peritoneal surface malignancy: experience with 1,000 patients. J Am Coll Surg.

[32] Van der Speeten K, Stuart OA, Chang D, Mahteme H, Sugarbaker PH (2011). Changes induced by surgical and clinical factors in the pharmacology of intraperitoneal mitomycin C in 145 patients with peritoneal carcinomatosis. Cancer Chemother Pharmacol.

[33] Turaga K, Levine E, Barone R, Sticca R, Petrelli N, Lambert L (2014). Consensus guidelines from The American Society of Peritoneal Surface Malignancies on standardizing the delivery of hyperthermic intraperitoneal chemotherapy (HIPEC) in colorectal cancer patients in the United States. Ann Surg Oncol.

[34] Chua TC, Yan TD, Smigielski ME, Zhu KJ, Ng KM, Zhao J (2009). Long-term survival in patients with pseudomyxoma peritonei treated with cytoreductive surgery and perioperative intraperitoneal chemotherapy: 10 years of experience from a single institution. Ann Surg Oncol.

[35] Elias D, Gilly F, Quenet F, Bereder JM, Sideris L, Mansvelt B (2010). Pseudomyxoma peritonei: a French multicentric study of 301 patients treated with cytoreductive surgery and intraperitoneal chemotherapy. Eur J Surg Oncol.

[36] Saxena A, Yan TD, Chua TC, Morris DL (2010). Critical assessment of risk factors for complications after cytoreductive surgery and perioperative intraperitoneal chemotherapy for pseudomyxoma peritonei. Ann Surg Oncol.

[37] Chua TC, Liauw W, Zhao J, Morris DL (2011). Upfront compared to delayed cytoreductive surgery and perioperative intraperitoneal chemotherapy for pseudomyxoma peritonei is associated with considerably lower perioperative morbidity and recurrence rate. Ann Surg.

[38] Youssef H, Newman C, Chandrakumaran K, Mohamed F, Cecil TD, Moran BJ (2011). Operative findings, early complications, and long-term survival in 456 patients with pseudomyxoma peritonei syndrome of appendiceal origin. Dis Colon Rectum.

[39] Dayal S, Taflampas P, Riss S, Chandrakumaran K, Cecil TD, Mohamed F (2013). Complete cytoreduction for pseudomyxoma peritonei is optimal but maximal tumor debulking may be beneficial in patients in whom complete tumor removal cannot be achieved. Dis Colon Rectum.

[40] Robella M, Vaira M, Marsanic P, Mellano A, Cinquegrana A, Sottile A (2013). Treatment of pseudomyxoma peritonei with cytoreductive surgery and hyperthermic intraperitoneal chemotherapy (HIPEC): a single center experience. Minerva Chir.

[41] Sparks DS, Morris B, Xu W, Fulton J, Atkinson V, Meade B (2015). Cytoreductive surgery and heated intraperitoneal chemotherapy for peritoneal carcinomatosis secondary to mucinous adenocarcinoma of the appendix. Int Surg.

[42] Ansari N, Chandrakumaran K, Dayal S, Mohamed F, Cecil TD, Moran BJ (2016). Cytoreductive surgery and hyperthermic intraperitoneal chemotherapy in 1000 patients with perforated appendiceal epithelial tumours. Eur J Surg Oncol.

[43] Azzam AZ, Alyahya ZA, Wusaibie AAA, Amin TM (2017). Cytoreductive surgery and hyperthermic intraperitoneal chemotherapy in the management of pseudomyxoma peritonei: a single-center experience. Indian J Gastroenterol.

[44] Grotz TE, Royal RE, Mansfield PF, Overman MJ, Mann GN, Robinson KA (2017). Stratification of outcomes for mucinous appendiceal adenocarcinoma with peritoneal metastasis by histological grade. World J Gastrointest Oncol.

[45] Choudry HA, Pai RK, Shuai Y, Ramalingam L, Jones HL, Pingpank JF (2018). Impact of cellularity on oncologic outcomes following cytoreductive surgery and hyperthermic intraperitoneal chemoperfusion for pseudomyxoma peritonei. Ann Surg Oncol.

[46] Delhorme JB, Severac F, Averous G, Glehen O, Passot G, Bakrin N (2018). Cytoreductive surgery and hyperthermic intraperitoneal chemotherapy for pseudomyxoma peritonei of appendicular and extra-appendicular origin. Br J Surg.

[47] Lansom J, Alzahrani N, Liauw W, Morris DL (2016). Cytoreductive surgery and hyperthermic intraperitoneal chemotherapy for pseudomyxoma peritonei and appendix tumours. Indian J Surg Oncol.

[48] Vaira M, Cioppa T, Marco GD, Bing C, D'Amico S, D'Alessandro M (2009). Management of pseudomyxoma peritonei by cytoreduction+HIPEC (hyperthermic intraperitoneal chemotherapy): results analysis of a twelve-year experience. Vivo.

[49] Levine EA, Votanopoulos KI, Shen P, Russell G, Fenstermaker J, Mansfield P (2018). A multicenter randomized trial to evaluate hematologic toxicities after hyperthermic intraperitoneal chemotherapy with oxaliplatin or mitomycin in patients with appendiceal tumors. J Am Coll Surg.

[50] Chua TC, Liauw W, Zhao J, Morris DL (2013). Comparative analysis of perioperative intraperitoneal chemotherapy regimen in appendiceal and colorectal peritoneal carcinomatosis. Int J Clin Oncol.

[51] Huang Y, Alzahrani NA, Liauw W, Traiki TB, Morris DL (2017). Early postoperative intraperitoneal chemotherapy for low-grade appendiceal mucinous neoplasms with pseudomyxoma peritonei: is it beneficial?. Ann Surg Oncol.

[52] Huang Y, Alzahrani NA, Liauw W, Soudy H, Alzahrani AM, Morris DL (2017). Early postoperative intraperitoneal chemotherapy is associated with survival benefit for appendiceal adenocarcinoma with peritoneal dissemination. Eur J Surg Oncol.

[53] Lam JY, McConnell YJ, Rivard JD, Temple WJ, Mack LA (2015). Hyperthermic intraperitoneal chemotherapy + early postoperative intraperitoneal chemotherapy versus hyperthermic intraperitoneal chemotherapy alone: assessment of survival outcomes for colorectal and high-grade appendiceal peritoneal carcinomatosis. Am J Surg.

[54] Tan GH, Ong WS, Chia CS, Tham CK, Soo KC, Teo MC (2016). Does early post-operative intraperitoneal chemotherapy (EPIC) for patients treated with cytoreductive surgery and hyperthermic intraperitoneal chemotherapy (HIPEC) make a difference?. Int J Hyperthermia.

[55] ICARuS Post-operative Intraperitoneal Chemotherapy (EPIC) and Hyperthermic Intraperitoneal Chemotherapy (HIPEC) After Optimal Cytoreductive Surgery (CRS) for Neoplasms of the Appendix, Colon or Rectum With Isolated Peritoneal Metastasis (ICARuS). https://www.clinicaltrials.gov/ct2/show/NCT01815359.

[56] Baratti D, Kusamura S, Nonaka D, Cabras AD, Laterza B, Deraco M (2009). Pseudomyxoma peritonei: biological features are the dominant prognostic determinants after complete cytoreduction and hyperthermic intraperitoneal chemotherapy. Ann Surg.

[57] Blackham AU, Swett K, Eng C, Sirintrapun J, Bergman S, Geisinger KR (2014). Perioperative systemic chemotherapy for appendiceal mucinous carcinoma peritonei treated with cytoreductive surgery and hyperthermic intraperitoneal chemotherapy. J Surg Oncol.

[58] Bijelic L, Kumar AS, Stuart OA, Sugarbaker PH (2012). Systemic chemotherapy prior to cytoreductive surgery and HIPEC for carcinomatosis from appendix cancer: impact on perioperative outcomes and short-term survival. Gastroenterol Res Pract.

[59] Lieu CH, Lambert LA, Wolff RA, Eng C, Zhang N, Wen S (2012). Systemic chemotherapy and surgical cytoreduction for poorly differentiated and signet ring cell adenocarcinomas of the appendix. Ann Oncol.

[60] Van Sweringen HL, Hanseman DJ, Ahmad SA, Edwards MJ, Sussman JJ (2012). Predictors of survival in patients with high-grade peritoneal metastases undergoing cytoreductive surgery and hyperthermic intraperitoneal chemotherapy. Surgery..

[61] Raghav KP, Shetty AV, Kazmi SM, Zhang N, Morris J, Taggart M (2013). Impact of molecular alterations and targeted therapy in appendiceal adenocarcinomas. Oncologist.

[62] Turner KM, Hanna NN, Zhu Y, Jain A, Kesmodel SB, Switzer RA (2013). Assessment of neoadjuvant chemotherapy on operative parameters and outcome in patients with peritoneal dissemination from high-grade appendiceal cancer. Ann Surg Oncol.

[63] Milovanov V, Sardi A, Ledakis P, Aydin N, Nieroda C, Sittig M (2015). Systemic chemotherapy (SC) before cytoreductive surgery and hyperthermic intraperitoneal chemotherapy (CRS/HIPEC) in patients with peritoneal mucinous carcinomatosis of appendiceal origin (PMCA). Eur J Surg Oncol.

[64] Spiliotis J, Kopanakis N, Efstathiou E, Vassiliadou D, Argiriou O, Rogdakis A (2017). Perioperative systemic chemotherapy for peritoneal mucinous appendiceal carcinomas treated with cytoreductive surgery & HIPEC. J BUON.

[65] Asare EA, Compton CC, Hanna NN, Kosinski LA, Washington MK, Kakar S (2016). The impact of stage, grade, and mucinous histology on the efficacy of systemic chemotherapy in adenocarcinomas of the appendix: analysis of the National Cancer Data Base. Cancer.

[66] Schomas DA, Miller RC, Donohue JH, Gill S, Thurmes PJ, Haddock MG (2009). Intraperitoneal treatment for peritoneal mucinous carcinomatosis of appendiceal origin after operative management: long-term follow-up of the Mayo Clinic experience. Ann Surg.

[67] Cummins KA, Russell GB, Votanopoulos KI, Shen P, Stewart JH, Levine EA (2016). Peritoneal dissemination from high-grade appendiceal cancer treated with cytoreductive surgery (CRS) and hyperthermic intraperitoneal chemotherapy (HIPEC). J Gastrointest Oncol.

