# Supplementary material for: Consensuses and controversies on pseudomyxoma peritonei: a review of the published consensus statements and guidelines
Source: Orphanet J Rare Dis. 2021 Feb 13;16:85. doi: 10.1186/s13023-021-01723-6 (PMC7881689; doi:10.1186/s13023-021-01723-6)
Supplement: Supplementary file 1 — Additional file 1. Classification of common adverse events during PMP perioperative period. [file 13023_2021_1723_MOESM1_ESM.docx]

| **Supplementary Table S1 Classification of common adverse events during PMP perioperative period** | | | | |
| --- | --- | --- | --- | --- |
| **Organ system** | **Adverse events classification and intervention measures** | | | |
|  | **I**  **(**Asymptomatic and self-limiting) | **II**  (Symptomatic and medical treatment) | **III**  **(**Invasive intervention**)** | **IV**  **(**ICU care Secondary surgery**)** |
| **Gastrointestinal system** |  |  |  |  |
| Anastomotic failure | Subclinical, afebrile, radiologic diagnosis | Antibiotics, febrile | Percutaneous drainage | Reoperation |
| Fistula | Subclinical, afebrile, radiologic diagnosis | Antibiotics, febrile | Percutaneous drainage | Reoperation |
| Pancreatic fistula | Elevated enzymes in drains | TPN + somatostatin | Percutaneous drainage | Reoperation |
| Pancreatitis | Elevated enzyme | Ranson’s score ≤3 | Ranson’s score 4 - 6 | Reoperation |
| Bile leak | Bile only in drain | Bile in drain, febrile | Percutaneous drainage | Reoperation |
| Chyle leak | Transient | Prolonged 1 week | Stop before discharge | Continued outside the hospital |
| Prolonged ileus | N/G | N/G >2 weeks | N/G >3 weeks | Persist past hospital  discharge |
| Small bowel obstruction | Abdominal pain | Abdominal pain, N/G reinsertion | Repeat radiologic studies | Reoperation |
| Hartmann pouch leak | Afebrile | Antibiotic, febrile | Percutaneous drainage | Reoperation |
| Enterostomy tube | Skin irritation at entrance site | Tube replaced on floor | Interventional radiology | Abscess formation, surgical drainage |
| Oral pain/ulceration | Soreness/erythema | Erythema, ulcers, can eat solids | Ulcers; requires liquid diet only | Alimentation not possible |
| Nausea/vomiting | Transient vomiting | Vomiting, anti-emetics | Vomiting, IV therapy | Vomiting, surgical intervention |
| Diarrhea | Transient <2 days | Tolerable, but >2 days | Intolerable, IV therapy | Dehydration prolonged IV therapy |
| Ascites | Mild | Fluid restriction | Symptomatic, percutaneous tap | Compromising vital function, ICU care |
| **Pulmonary System** | | | | |
| Respiratory distress | Mild symptom | Oxygen therapy or medications | Endotracheal intubation | Tracheostomy |
| Pleural effusion | Asymptomatic | Diuretics required | Thoracentesis required | Compromised, chest tube insertion |
| Pneumonia | Mild symptoms | Antibiotics and respiratory therapy | Bronchoscopy | Intubation required |
| Acute respiratory distress | Mild symptoms | Moderate respiratory support | Prolonged respiratory support | Tracheostomy, ICU |
| Chest tube removal | Radiologic diagnosis | Heimlich valve | Chest tube insertion | Tension pneumothorax |
| Pneumothorax | <10% | >10% | Heimlich valve | Chest tube reinserted |
| **Intravenous Catheter** | | | | |
| Line sepsis | Entrance site only | Positive cultures, elective line removal | Bacteremia, urgent line removal | Septic shock, ICU care |
| Line- thrombosis | Swelling, minor | Swelling, moderate elective line removal | Anticoagulation, line removal | Clot lysis |
| Pneumothorax | Radiology (+) only | Oxygen therapy, in-hospital observation | Chest tube insertion | Tension pneumothorax |
| TPN intolerance | Mild | Moderate | Severe | Discontinuation |
| **Cardiovascular System** | | | | |
| Arrhythmia | Sinus tachycardia | Medical therapy | Interventions | ICU care |
| Hypotension | Orthostatic | IV therapy | Vasopressors | ICU care |
| Ischemia | SAP | UAP | NSTEMI | STEMI |
| Pulmonary embolism | Asymptomatic | Anticoagulation | Vein filter | ICU care/surgery |
| Thrombophlebitis | Cellulitis only | Lymphangitis | Bacteremia | Septicemia |
| Venous thrombosis | Extremity only | Anticoagulant | Vein filter | With PE, ICU care |
| Pulmonary edema | Fluid restriction | Diuretics | Pressure ventilation | Intubation, ICU care |
| 5-fluorouracil toxicity | Discontinue 5-FU | Diuretics | CHF | ICU care |
| **Genitourinary System** | | | | |
| Urinary tract infection | Asymptomatic bacteriuria | Bacteriuria, elevated temperature or WBC | Bacteriuria, positive blood cultures | Urosepsis, ICU care |
| Urine leak | Leak detected | Catheterization | Invasive intervention | Reoperation |
| Vaginal bleed | Staining only | Drop in Hct/Hgb | Blood replacement | Reoperation |
| Acute renal failure | Abnormal creatinine | Respond to diuretics | Fluid management required | Dialysis required |
| **Hematologic System** | | | | |
| Leukocytes/mm3 | 2000 - 3000 | 1000 - 2000 | 0 - 1000 | Sepsis |
| Platelets (1000) | 50 - 99 | 10 - 50 | 0 - 10 | Bleeding |
| Anemia/bleeding | No replacement | ≤4 units | >4 units | Reoperation |
| **Neurologic system** | | | | |
| Mental status | Transient lethargy | Somnolence <50% of waking hours | Somnolence >50% of waking hour | Coma, ICU care |
| Orientation/intellect | Mild confusion | Mild disorientation, but able to care for self | Disorientation, unable to care for self | Grossly disoriented, combative, psychotic |
| Stroke | TIA | RIND | Stroke unit care | ICU care |
| Neuropathy/nerve paralysis | Transient symptoms | Persistent symptoms | Functional deficit resolved before discharge | Functional deficit after discharge |
| **Infection** | | | | |
| Intra-abdominal | Minimal symptoms | Prolonged antibiotics | Percutaneous drainage | Reoperation |
| Wound | Cellulitis and swelling | Antibiotics | Open wound | Reoperation |
| **Skin/abdominal wall** |  |  |  |  |
| Allergic | Urticaria | Bronchospasm | Bronchospasm requiring medication | Anaphylaxis with ICU care |
| Wound dehiscence | Skin sutures | Fascia defect <6 cm | Fascia defect >6 cm | Reoperation |
| **Others** | NA | NA | NA | NA |
| Abbreviations: TPN, total parenteral nutrition; ICU: intensive care unit; SAP: stable angina pectoris; UAP: unstable angina pectoris; NSTEMI, non-ST segment elevation acute myocardial infarction; STEMI, ST segment elevation acute myocardial infarction; PE, pulmonary embolism; CHF, chronic heart failure; TIA, transient ischemic attack; RIND, reversible ischemic neurological deficit; NA, not applicable. | | | | |
